# The dynamic relationship between physical activity and psychological well-being in Chinese older adults: a longitudinal cross-lagged panel network analysis

**DOI:** 10.3389/fpubh.2025.1736019

**Published:** 2026-01-06

**Authors:** Luyao Xiang, Jing Yang, Hao Gou, Chang Hu

**Affiliations:** 1 Zhuhai Campus, Zunyi Medical University, Zhuhai, China; 2 School of Psychology and Sociology, Mianyang Normal University, Mianyang, China; 3 College of Physical Education, Qiannan Normal University for Nationalities, Duyun, China; 4 College of Physical Education, Jiangxi Normal University, Nanchang, China

**Keywords:** cross-lagged panel network analysis, older adults, longitudinal study, physical activity, psychological well-being

## Abstract

**Background:**

With the ongoing deepening of population aging in China, the psychological well-being of older adults people has become an important indicator for measuring social health development. Physical activity, as an interventionable health behavior, has a complex relationship with psychological well-being. However, the dynamic interaction mechanisms between the two have not been systematically revealed.

**Methods:**

This study employed a longitudinal design, tracking 967 older adults individuals from Guiyang and Duyun in Guizhou Province and Nanchang in Jiangxi Province between February and April 2024 (T1) and February and April 2025 (T2). Using cross-lagged panel network analysis (CLPN), which integrates both cross-sectional and longitudinal network models, we examined the temporal predictive relationships between physical activity and the multidimensional aspects of psychological well-being.

**Results:**

(1) The network connection density between physical activity and psychological well-being increased from 56.84% at T1 to 60.00% at T2, indicating a continuous enhancement of system associations; (2) The cross-sectional network revealed that the core association shifted from “personal growth—openness” (*r* = 0.488) to “autonomous value—self-assurance” (*r* = 0.393); (3) The cross-lagged network demonstrated that physical activity has a predictive effect on psychological well-being, with the frequency of exercise significantly predicting autonomous values (β = 0.166); (4) “Meaning in life” plays both a driving and bridging role in the system and is a key target for interventions.

**Conclusion:**

Physical activity and psychological well-being in older adults individuals constitute a dynamic, bidirectional promoting system, with the path of influence evolving in stages from social support to personal growth. The findings provide a theoretical basis and practical guidance for developing staged and precise health promotion programs for the older adults.

## 1 Introduction

In the context of a quietly aging population, “old age” has shifted from being a marginal issue to the center of social discourse (1, 2). As the silver wave continues to push up the social dependency ratio, the first concerns people often see are the pension gap, the shortage of caregivers, and the compounded burden of chronic diseases (3, 4). However, another more hidden cost is frequently overlooked—the systematic loss of mental health in later life stages (5). Loneliness, loss of value, and lack of meaning ripple silently, gradually amplified by a series of life events such as retirement, bereavement (6, 7), and declining physical function, making longevity, in some ways, a challenge rather than a social achievement (8). In China, with the deepening of population aging, more and more older adults individuals face challenges in life after retirement. According to current Chinese policies, the legal retirement age is 60 for men and 55 for women. Therefore, the group of participants aged between 60 and 70 years in our study may be closely related to the characteristics of life after retirement (9). Therefore, focusing on the psychological well-being of the older adults has transcended mere ethical care and has become a core indicator of whether a society can achieve aging with grace (10, 11). Psychological well-being is far from a fleeting positive emotion; it is a multidimensional construct that encompasses the pursuit of life meaning, self-acceptance, control over the environment, positive interpersonal relationships, and experiences of continuous growth (12). It determines whether older adults individuals can still maintain vitality, value, and dignity in the face of inevitable physiological decline (13, 14). For this reason, systematically clarifying the key pathways and internal mechanisms for improving older adults psychological well-being has become a fundamental issue that aging societies must address.

Among the many influencing factors, physical activity is considered a highly promising intervention due to its universality, low cost, and multiple benefits (15). From a theoretical perspective, physical activity and mental health share a profound neurobiological foundation (16). Regular physical exertion effectively regulates neurotransmitters and brain regions closely associated with emotions and cognition, thereby laying a physiological foundation for the enhancement of psychological well-being (17). At the same time, according to self-determination theory, successfully engaging in and autonomously controlling physical activity can directly satisfy an individual’s basic psychological needs for competence, relatedness, and autonomy, thus becoming an intrinsic source that nurtures psychological well-being (18). A large body of empirical evidence consistently shows that older adults individuals who actively participate in activities such as walking, Tai Chi, or square dancing generally exhibit higher life satisfaction and a stronger sense of meaning in life (19–21). However, the relationship between the two is far from a simple, unidirectional influence (22). According to the broaden-and-build theory, higher psychological well-being can help individuals accumulate psychological resources, expand their willingness and ability to act, thus enabling them to engage in physical activity more actively and persistently (22). An older adults person who feels a sense of purpose in life is also more likely to view maintaining physical activity as an important way to achieve a valuable life (23–25). This suggests that physical activity and psychological well-being constitute a complex, bidirectional, mutually beneficial dynamic system (26–28).

However, existing research still has limitations in revealing the fine-grained dynamic mechanisms between the two (29). While the majority of cross-sectional studies can confirm the associations between variables, they fail to clarify the directionality and temporal dynamics of their mutual influences (30). More critically, traditional variable-centered analytical methods often treat psychological well-being as a single latent variable, which obscures the unique roles that its different dimensions may play (31). To address these limitations, we introduce a network analysis framework, which moves beyond the traditional limitations of CLPM. This approach allows for a more nuanced examination of psychological well-being by treating it as a multidimensional construct, capturing the temporal dynamics and bidirectional relationships between physical activity and the specific dimensions of well-being over time. By incorporating both cross-sectional and longitudinal data, network analysis reveals how different dimensions of psychological well-being interact with physical activity, offering unique theoretical insights that CLPM cannot provide (32). This method integrates both cross-sectional and longitudinal data, aiming to unravel the complex interaction system between variables (33, 34). Specifically, the analytical framework consists of two core components: First, by constructing a cross-sectional network, this method can depict the unique synchronous association patterns between physical activity and the various dimensions of psychological well-being at a given time point, and calculate centrality indicators to identify the variables that hold central positions and exert stronger influence in the network at that moment. This reveals the instantaneous association structure between variables and their stability and evolution over time (35). Second, and most innovatively, is the construction of a cross-lagged network. Based on longitudinal data, this network systematically examines the cross-time reciprocal predictive relationships between all variables (36). It not only identifies the overall directionality of predictive relationships—whether physical activity predominantly predicts subsequent changes in psychological well-being, or psychological well-being more strongly drives subsequent levels of physical activity, or whether there are significant bidirectional predictive pathways—but also precisely dissects this temporal predictive effect between each pair of variables, thus revealing the specific paths of dynamic interactions (37). On this basis, by calculating directed centrality indicators such as InStrength and OutStrength, it becomes possible to precisely pinpoint two key nodes within the system (38, 39). This study deepens the analysis from static associations to dynamic predictions, from overall structure to specific paths, allowing it to go beyond traditional association proof and truly depict the operational mechanisms of a dynamic system, thus providing key evidence for identifying the most effective intervention targets.

## 2 Methods

### 2.1 Participants and procedures

This study employed a prospective longitudinal design and used purposive sampling to recruit older adults participants who met the following inclusion criteria from communities, nursing homes, and senior universities in Guiyang and Duyun cities in Guizhou Province, as well as Nanchang city in Jiangxi Province: (1) Age between 60 and 80 years; (2) No severe cognitive impairments, able to understand and complete the questionnaires; (3) Residing in the specific cities or communities defined for this study to ensure regional representativeness. In each setting, local community workers, nursing home administrators, and senior university staff assisted in identifying potentially eligible older adults and informing them about the study. Recruitment information was disseminated through posters, notice-board announcements, and brief on-site presentations, and interested individuals were approached face to face by trained research assistants. The research assistants provided a concise introduction to the study aims and procedures, conducted an initial eligibility screening based on the inclusion criteria, and then invited those who were eligible to participate. Older adults who agreed to take part signed a written informed consent form before completing the questionnaire.

The baseline survey was conducted from February to April 2024, with a total of 1,068 valid questionnaires collected. The follow-up survey was completed between February and April 2025, with 967 older adults participants who participated in both rounds of the survey, meeting the above inclusion criteria, and were included in the final analysis. To address missing data, this study employed multiple imputation based on chained equations in the R programming environment, using the mice package to impute missing values (40). To further examine potential biases due to sample attrition, we compared participants who completed both rounds of the survey with those who only completed the baseline survey. Independent samples *t*-tests showed no significant differences between the two groups in terms of gender (*t* = 0.417, *p* > 0.05), marital status (*t* = −0.878, *p* > 0.05), education level (*t* = −1.202, *p* > 0.05), annual income (*t* = 0.401, *p* > 0.05), baseline physical activity levels (*t* = −1.960, *p* > 0.05), or psychological well-being (*t* = 1.920, *p* > 0.05). These results indicate that sample attrition did not lead to structural bias.

The final sample included 967 older adults individuals who completed both rounds of assessments, with their basic characteristics shown in Table 1. Among them, 372 were male (38.5%) and 595 were female (61.5%). The age distribution was primarily between 60 and 70 years, with 536 individuals (55.4%), followed by 321 individuals (33.2%) aged 71 to 80, and 110 individuals (11.4%) aged over 80. In terms of education level, 108 individuals (11.2%) were illiterate or semi-illiterate, 297 individuals (30.7%) had completed primary school, 398 individuals (41.2%) had completed middle school, and 164 individuals (17.0%) had completed high school or higher. Regarding marital status, 746 individuals (77.1%) had a spouse, while 221 individuals (22.9%) were without a spouse. As for annual income, 593 individuals (61.3%) had an income below 7,500 RMB, while 374 individuals (38.7%) had an income above 7,500 RMB.

**Table 1:** Basic characteristics of the sample.

Variable	Sort	Frequency	Scale
Sex	Male	372	38.5%
	Female	595	61.5%
Education level	Illiterate or semi-illiterate	108	11.2%
	Primary school	297	30.7%
	Middle school	398	41.2%
	High school or above	164	17.0%
Age	60–70 years	536	55.4%
	71–80 years	321	33.2%
	Over 80 years	110	11.4%
Marital status	With a spouse	746	77.1%
	Without a spouse	221	22.9%
Annual income	Less than 7,500 RMB	593	61.3%
	More than 7,500 RMB	374	38.7%

### 2.2 Measuring tools

#### 2.2.1 Psychological well-being scale

This study systematically adapted, revised, and culturally adjusted the Psychological Well-Being Scale based on the theoretical framework and original measurement system proposed by psychologist Li (41). The revision process strictly followed internationally accepted guidelines for cross-cultural scale adaptation, and it involved the following key stages (42): First, during the translation and back-translation phase, two bilingual psychological researchers independently completed the forward translation of the scale. After comprehensive discussion within the research team, a preliminary Chinese version was formed. Subsequently, a native English speaker, who had not been exposed to the original scale, was invited to perform the back-translation. The core research team compared the back-translated version with the original text to ensure conceptual equivalence and accuracy. Next, a committee composed of experts in health psychology, gerontology, and linguistics was formed to review and culturally adapt the translated version. The experts reviewed the semantic equivalence, cultural appropriateness, and comprehensibility of each item for the older adults population. Some expressions were localized to better fit the life context of Chinese older adults individuals. Subsequently, a pre-test was conducted within the research group. Through cognitive interviews and pilot testing of the questionnaire, the researchers gained insights into how older adults participants understood the items. Based on this, ambiguous wording was further optimized to ensure the scale had both good face validity and content validity. After this systematic process, the final official scale included 18 items, covering six dimensions: autonomy, environmental mastery, personal growth, positive interpersonal relationships, life purpose, and self-acceptance. The scale used a 6-point Likert scoring system (1 = strongly disagree to 6 = strongly agree), with higher scores indicating higher levels of psychological well-being.

To test its psychometric properties, this study conducted a systematic validation using two waves of longitudinal data. The results of confirmatory factor analysis showed that the six-factor model demonstrated good goodness-of-fit at both time points (specific indicators are provided in Supplementary Table S1), confirming that the scale has ideal structural validity. Regarding reliability, the Cronbach’s α coefficients for the total scale at T1 and T2 were 0.894 and 0.914, respectively. The internal consistency coefficients for each dimension met the psychometric standards (see Supplementary Table S2), indicating that the Chinese version of the scale has good measurement stability and reliability among the older adults population in China.

#### 2.2.2 Physical exercise scale

This study used the Physical Activity Rating Scale revised by Liang Deqing to assess the physical activity levels of older adults individuals (43). The scale quantifies physical activity behaviors across three dimensions: exercise intensity, exercise frequency, and duration of each exercise session, with one item for each dimension, using a five-point rating system. In this study, the Cronbach’s α coefficients for the scale at T1 and T2 were 0.889 and 0.844, respectively, indicating good internal consistency reliability at both measurement time points.

### 2.3 Data analysis

#### 2.3.1 Preliminary analysis

The data analysis in this study was divided into two main stages: preliminary analysis and network modeling. First, descriptive statistics were conducted on the scores of physical activity and psychological well-being across the dimensions at both T1 and T2. Subsequently, independent samples *t*-tests were used to compare the differences between older adults individuals who dropped out at T2 and those who continued participating, with respect to core variables, in order to assess potential systematic biases due to sample attrition. Based on this, one-way analysis of variance (ANOVA) and independent samples *t*-tests were used to identify demographic variables with significant differences, which were then included as covariates in the subsequent network analysis model. Additionally, Harman’s single-factor test was applied to check for common method bias in the data at both T1 and T2. All of the above analyses were performed using SPSS 27.0.

#### 2.3.2 Network analysis

Network analysis was conducted in the R 4.3.2 environment, including both cross-sectional network and cross-lagged panel network analysis. First, cross-sectional networks were constructed separately based on the data from T1 and T2. A mixed graphical model was used for estimation, and the EBICglasso algorithm was employed for model regularization to optimize the network structure, with the hyperparameter γ of the extended Bayesian information criterion (EBIC) set to 0.5 (44). For centrality metrics, expected influence was chosen as the measure of node impact, as it provides better stability when dealing with mixed polarity networks.

Furthermore, this study used cross-lagged panel network analysis (CLPN) to examine the predictive relationships between physical activity and psychological well-being over time. CLPN integrates autoregressive paths and cross-lagged paths, allowing for the assessment of how each variable is influenced by the prior levels of other variables while controlling for its own previous levels. The modeling was implemented using the glmnet package in R, which employs regularized regression (specifically lasso regression) to estimate the network edges. The lambda parameter, which controls the strength of the regularization, was selected using 10-fold cross-validation to minimize mean squared error (MSE) and ensure optimal model performance. Covariates such as age and gender were included in the model as part of the predictor matrix, but they were not regularized. This allowed us to control for their effects while focusing on the dynamic interactions between physical activity and psychological well-being. To identify key nodes in the network, we calculated directed bridge centrality indicators, including in-strength expected influence and out-strength expected influence. These indicators measure how much a node is predicted by other nodes (in-strength) and its ability to predict other nodes (out-strength), helping to identify central variables within the network. Network visualization was carried out using the qgraph package in R, where nodes represent variables and edges represent the relationships between them. The results were displayed with a spring layout to visually reflect the strength and directionality of these relationships. This expanded explanation now provides a more complete description of the CLPN method, including the selection of the regularization parameter, the handling of covariates, and the calculation of centrality indicators, offering greater transparency and clarity regarding the methodology (45).

Finally, the Bootstrap sampling method was used to generate 1,000 iterations, and the 95% confidence intervals of edge weights were computed to assess the accuracy of the estimates. The stability of the centrality indicators was tested using the correlation stability coefficient (CS). A CS value greater than 0.25 is generally considered acceptable, and a CS greater than 0.5 indicates good stability (46).

## 3 Results

### 3.1 Common method bias test

This study employed Harman’s single-factor test to analyze common method bias. The results showed that in both time points, there were seven factors with eigenvalues greater than 1 (47). The first factor explained 35.45% of the variance at T1 and 37.52% at T2, both of which were below the 40% critical threshold. This indicates that there is no serious common method bias issue in the data of this study.

### 3.2 Descriptive analysis

To comprehensively describe the sample characteristics, Table 2 presents the descriptive statistics for the indicators of physical activity and psychological well-being at both time points. Based on this, one-way analysis of variance (ANOVA) and independent samples *t*-tests were conducted to examine the influence of demographic variables on the core study variables. The results indicated that gender, age, education level, marital status, and annual income all had significant effects on physical activity levels and psychological well-being scores. Therefore, in the subsequent network analysis, these variables were included as covariates in the model for control.

**Table 2:** Descriptive statistics for physical activity and psychological well-being indicators (M ± SD).

Variable	Item	Label	T1		T2	
			*M*	SD	*M*	SD
Physical activity	How intense is your exercise?	PA1	2.605	1.272	2.719	1.365
	How many minutes do you engage in the above-intensity physical activity at a time?	PA2	2.634	1.300	2.769	1.349
	How many times a month do you engage in the above-intensity physical activity?	PA3	2.601	1.289	2.730	1.364
Psychological well-being	My decisions are rarely influenced by others	PWB1	3.148	1.654	3.222	1.741
	I live according to my own beliefs and values, regardless of what others say	PWB2	3.090	1.634	3.208	1.714
	I am confident in my viewpoints, even if they differ from the majority	PWB3	3.134	1.615	3.263	1.698
	I am good at handling responsibilities in daily life	PWB4	3.154	1.652	3.274	1.683
	I am able to create a comfortable and satisfactory environment for myself	PWB5	3.115	1.627	3.222	1.702
	I am good at utilizing opportunities around me to achieve my goals	PWB6	3.203	1.621	3.242	1.698
	I believe life is a continuous process of learning, change, and growth	PWB7	3.202	1.637	3.237	1.698
	I am open to new things and new experiences	PWB8	3.175	1.649	3.311	1.744
	Over time, I can see myself making progress and maturing	PWB9	3.203	1.629	3.270	1.737
	I have warm, trusting, and mutually supportive close relationships	PWB10	3.190	1.567	3.355	1.667
	I know how to care for others and am willing to make sacrifices for my friends	PWB11	3.287	1.607	3.331	1.687
	When I need it, I can receive emotional support and help from my friends	PWB12	3.275	1.599	3.355	1.670
	My life has a clear sense of direction and purpose	PWB13	3.163	1.608	3.201	1.735
	I sometimes think about the meaning of life and my life’s mission	PWB14	3.243	1.609	3.326	1.686
	My daily life is meaningful and purposeful	PWB15	3.123	1.648	3.258	1.727
	I have a positive attitude toward myself and generally like who I am	PWB16	3.133	1.604	3.218	1.697
	I accept all aspects of my personality, including my flaws	PWB17	3.128	1.598	3.210	1.690
	In retrospect, I am satisfied with my life experiences	PWB18	3.172	1.608	3.186	1.681

### 3.3 Cross-sectional network analysis

**Figure 1:**
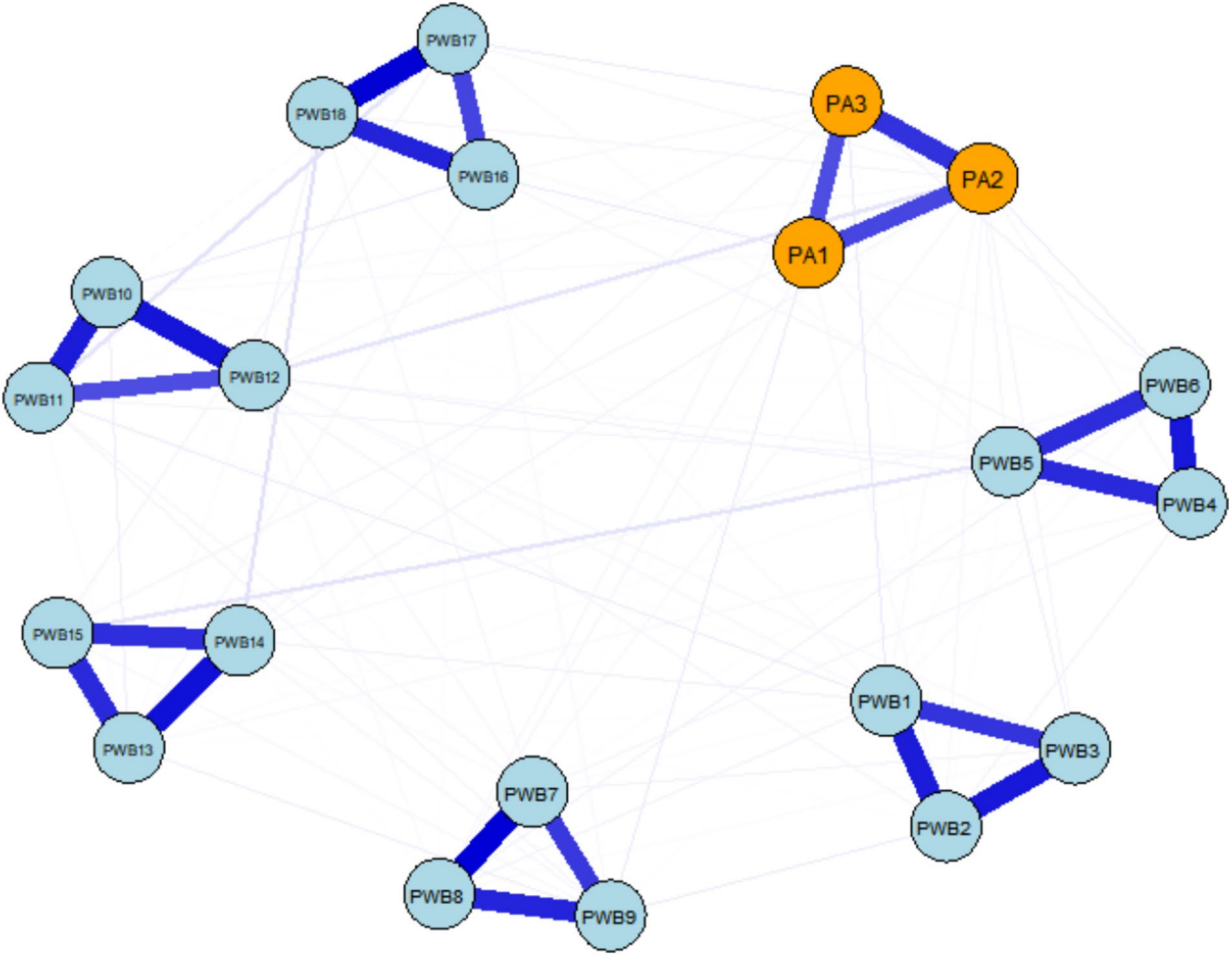
Cross-sectional network of physical activity and psychological well-being (T1). The blue solid lines in the figure represent positive associations between variables, while the red dashed lines represent negative associations. The thickness of the lines is proportional to the strength of the associations. The labels for each node and their specific meanings can be found in Table 2. The same applies to the figure below.

**Figure 2:**
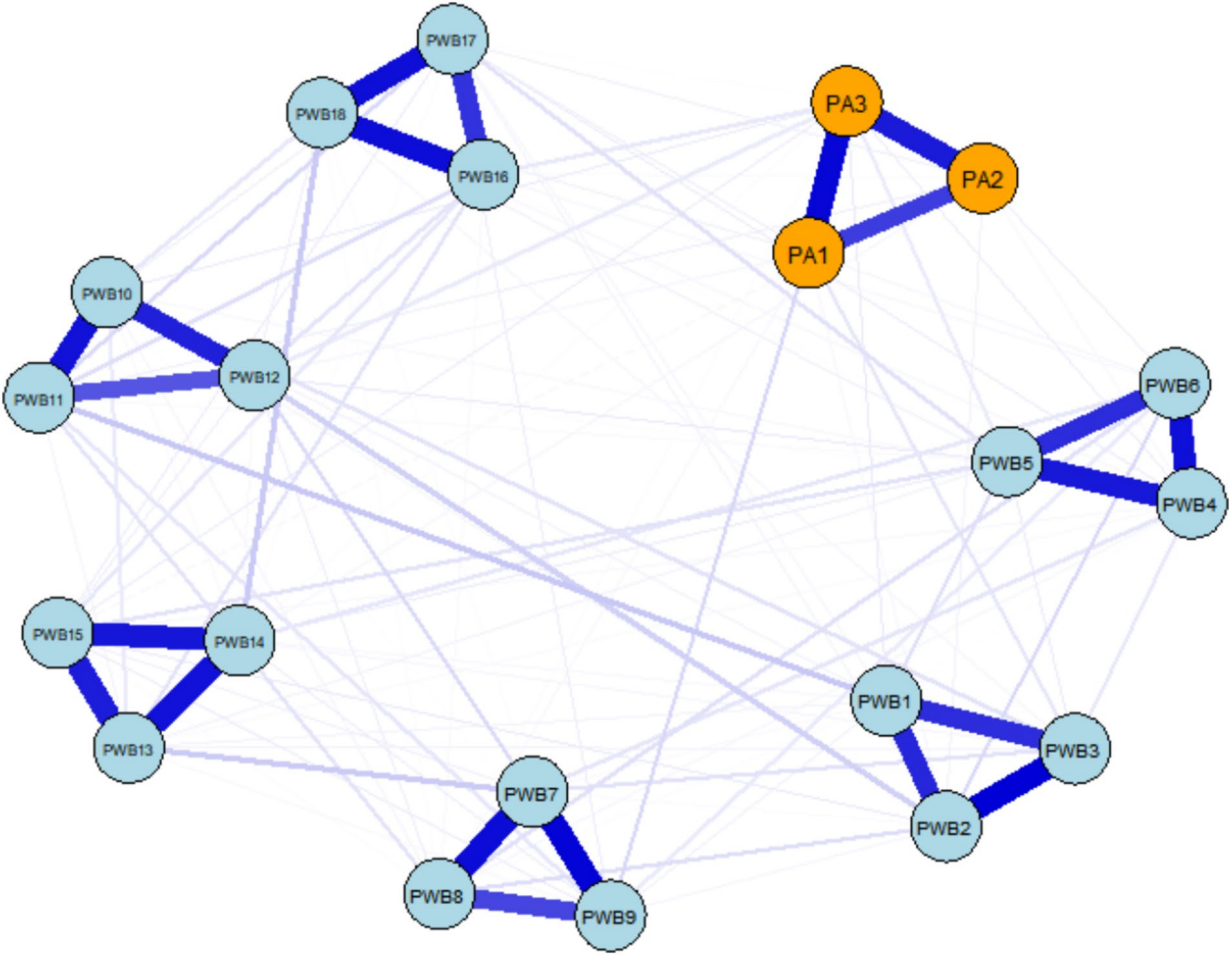
Cross-sectional network of physical activity and psychological well-being (T2).

The results of the cross-sectional network analysis showed that the network at the T1 time point consisted of 108 non-zero edges, with a network density of 0.568, indicating that 56.84% of possible connections were actually present. By the T2 time point, the complexity of the network increased, with the number of non-zero edges rising to 114, and the network density increasing to 0.600 (60.00%), reflecting an overall enhancement in connection strength. A comparison of the network structures between the two time points (Figures 1, 2) revealed a trend of strengthening internal associations over time. In the T1 network, the strongest association appeared within psychological well-being, specifically between “I believe life is a continuous process of learning, change, and growth” (PWB7) and “I am open to new things and new experiences” (PWB8) (*r* = 0.488). The most prominent cross-domain connection was between “How many minutes do you engage in the above-intensity physical activity at a time?” (PA2) and “When I need it, I can receive emotional support and help from my friends” (PWB12) (*r* = 0.048), suggesting a weak but identifiable link between physical activity and social support.

By T2, the strongest association in the network shifted to “I live according to my own beliefs and values, regardless of what others say” (PWB2) and “I am confident in my viewpoints, even if they differ from the majority” (PWB3) (*r* = 0.393), reflecting the stable covariance between individual autonomy and self-assurance. The key cross-domain edge at this point was between “How intense is your exercise?” (PA1) and “Over time, I can see myself making progress and maturing” (PWB9) (*r* = 0.061), indicating the emergence of a preliminary predictive relationship between exercise intensity and a sense of personal growth. The specific values of all the edges in the cross-sectional networks are provided in Supplementary Tables S3, S4. Overall, the network structure at both time points maintained stable connections between core psychological constructs and also demonstrated the gradual emergence of dynamic relationships between physical activity and the psychological well-being sub-systems.

**Figure 3:**
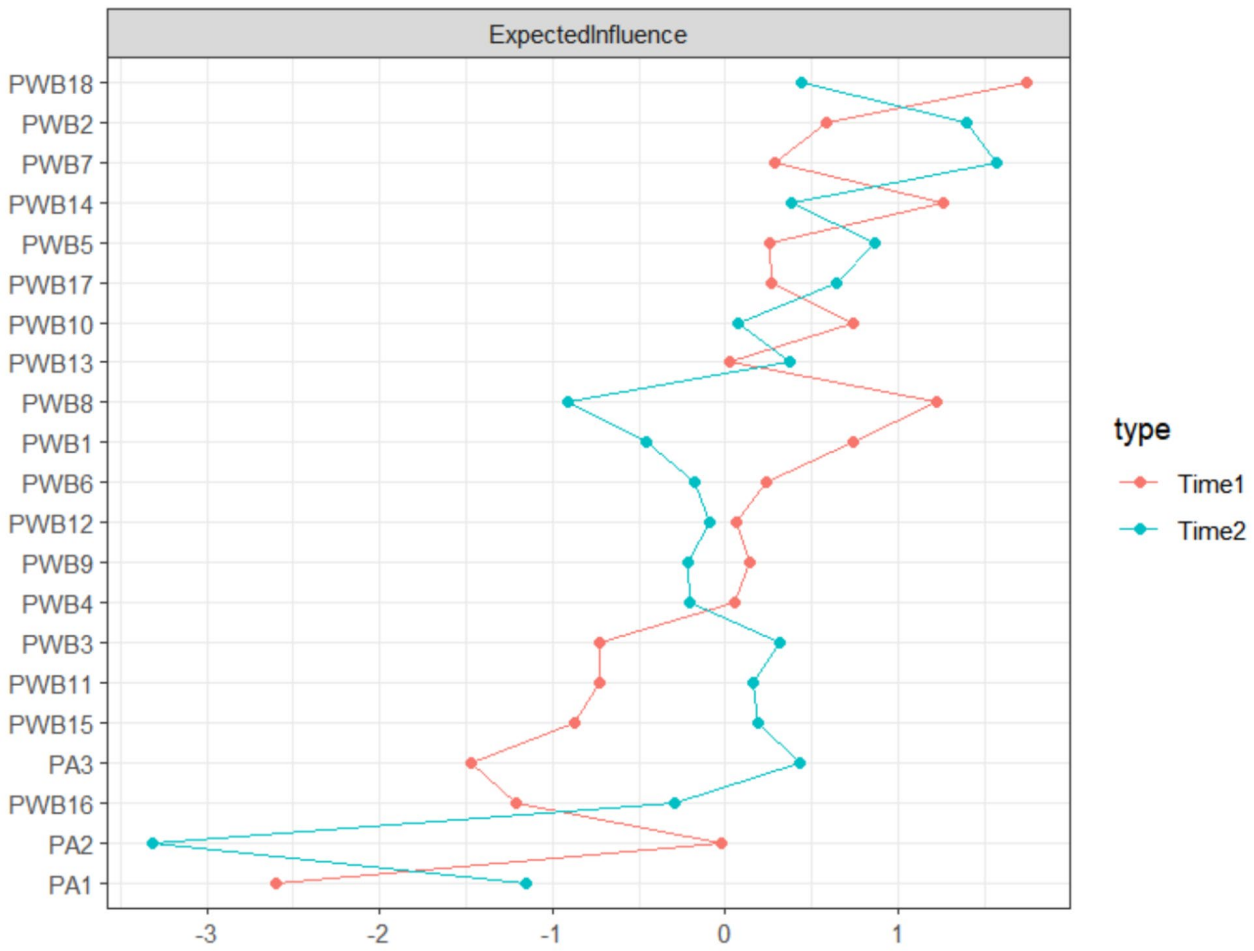
Centrality indicators (expected influence values) of physical activity and psychological well-being at T1 and T2.

Figure 3 shows the expected influence (EI) centrality values of the two cross-sectional networks at T1 and T2. At the T1 time point, the node with the highest centrality was “In retrospect, I am satisfied with my life experiences” (PWB18, EI = 1.740), indicating that life satisfaction had a broad influence within the system. By the T2 time point, the most influential node in the network shifted to “I believe life is a continuous process of learning, change, and growth” (PWB7, EI = 1.571), reflecting the core role of a growth mindset in the later network. Both of the most central nodes at T1 and T2 belonged to the psychological well-being dimension, reflecting its dominant position in the system structure. Specific centrality indicators for all nodes at both time points are detailed in Supplementary Tables S5, S6.

To assess the reliability of the cross-sectional network structure, this study used the Bootstrap sampling method to test the stability and accuracy of the T1 and T2 networks. The results showed that the 95% Bootstrap confidence intervals for the edge weights in both networks were relatively concentrated, indicating that the network estimates were overall stable. Regarding the correlation stability coefficients, in the T1 network, the CS coefficient for edges was 0.75, showing high stability; the eigenvector centrality was 0.517, reflecting good stability; the expected influence and strength centralities both had a CS of 0.361, which is within an acceptable range. In the T2 network, the stability of the indicators further improved: the CS for edges reached 0.75, representing the best stability; the eigenvector centrality was 0.672, demonstrating good stability; and the expected influence and strength centralities were 0.438, ranging from acceptable to good levels. All CS coefficients were above the minimum acceptable threshold of 0.25, indicating that the network centrality estimates had sufficient stability to support further analysis. The Bootstrap confidence intervals for the edge weights and the results of centrality difference tests are detailed in the supplementary materials.

### 3.4 Cross-lagged panel network analysis

**Figure 4:**
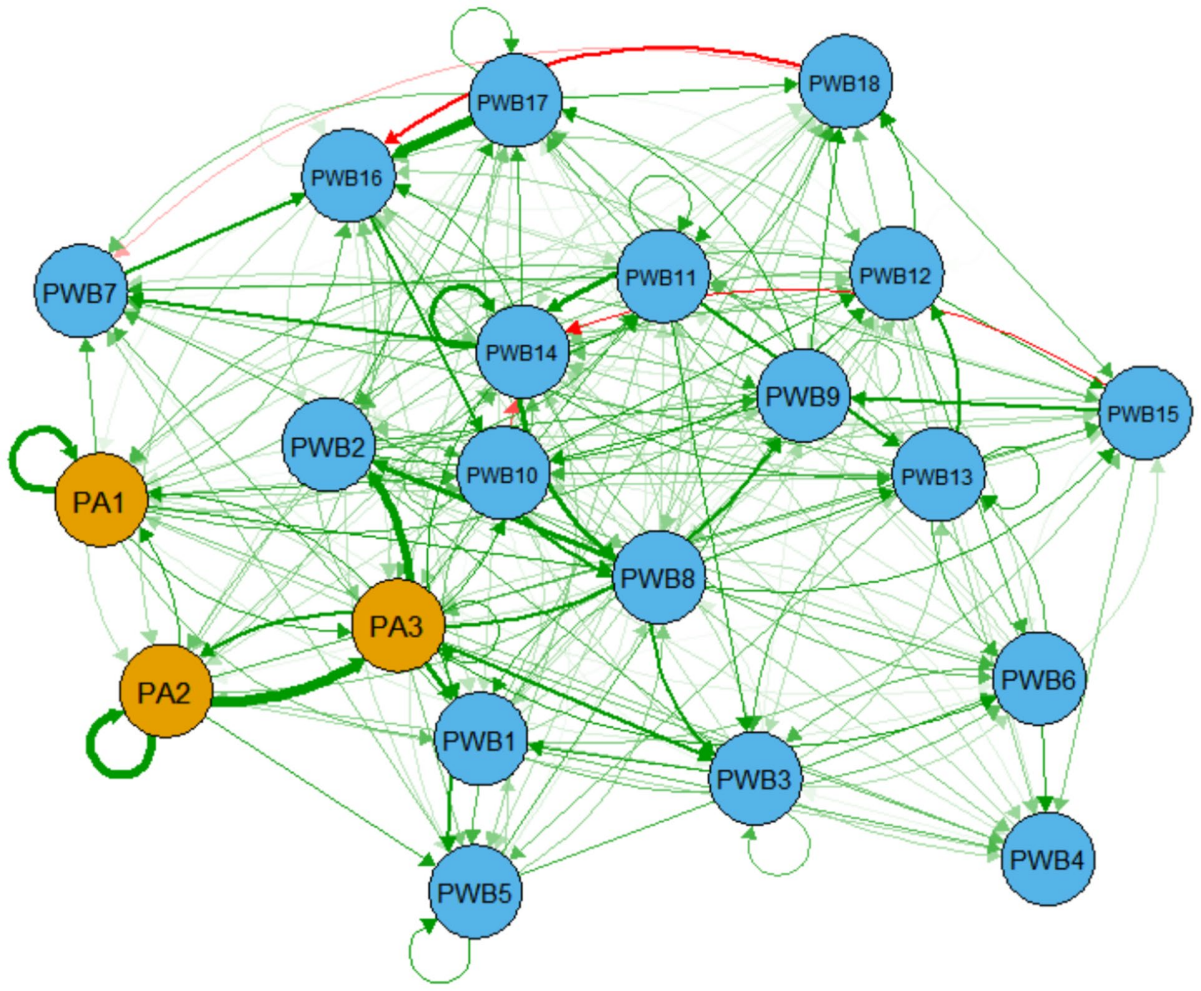
Cross-lagged panel network of physical activity and psychological well-being. The green solid arrows in the figure represent positive predictive effects, while the red dashed arrows represent negative predictive effects. The thickness of the lines is proportional to the standardized regression coefficients (β values), and the direction of the arrows indicates the direction of the predictive relationship. The node labels correspond to the variable names and specific item content, as detailed in Table 2.

The cross-lagged panel network included 166 non-zero edges, revealing the complex temporal interactions between physical activity and psychological well-being across various dimensions (Figure 4). Among all the predictive paths, the strongest cross-lagged effect was observed in the physical activity dimension, where “exercise duration” (PA2) had the most significant predictive effect on “exercise frequency” (PA3) (β = 0.187), indicating that the time invested in exercise can effectively predict the subsequent maintenance of exercise frequency. Within psychological well-being, “self-acceptance” (PWB17) exhibited a significant predictive effect on “self-affirmation” (PWB16) (β = 0.184), reflecting the role of self-acceptance in promoting overall self-evaluation. Importantly, the study also found a cross-domain predictive path from physical activity to psychological well-being, specifically with “exercise frequency” (PA3) having a significant positive predictive effect on “autonomous values” (PWB2) (β = 0.166), suggesting that regular exercise participation may enhance individuals’ sense of value autonomy. In terms of autoregressive effects, “exercise duration” (PA2) showed the strongest self-predictive effect (β = 0.152), demonstrating the relative stability of this behavior over time. The specific coefficients for these core predictive paths can be found in Supplementary Table S7.

**Figure 5:**
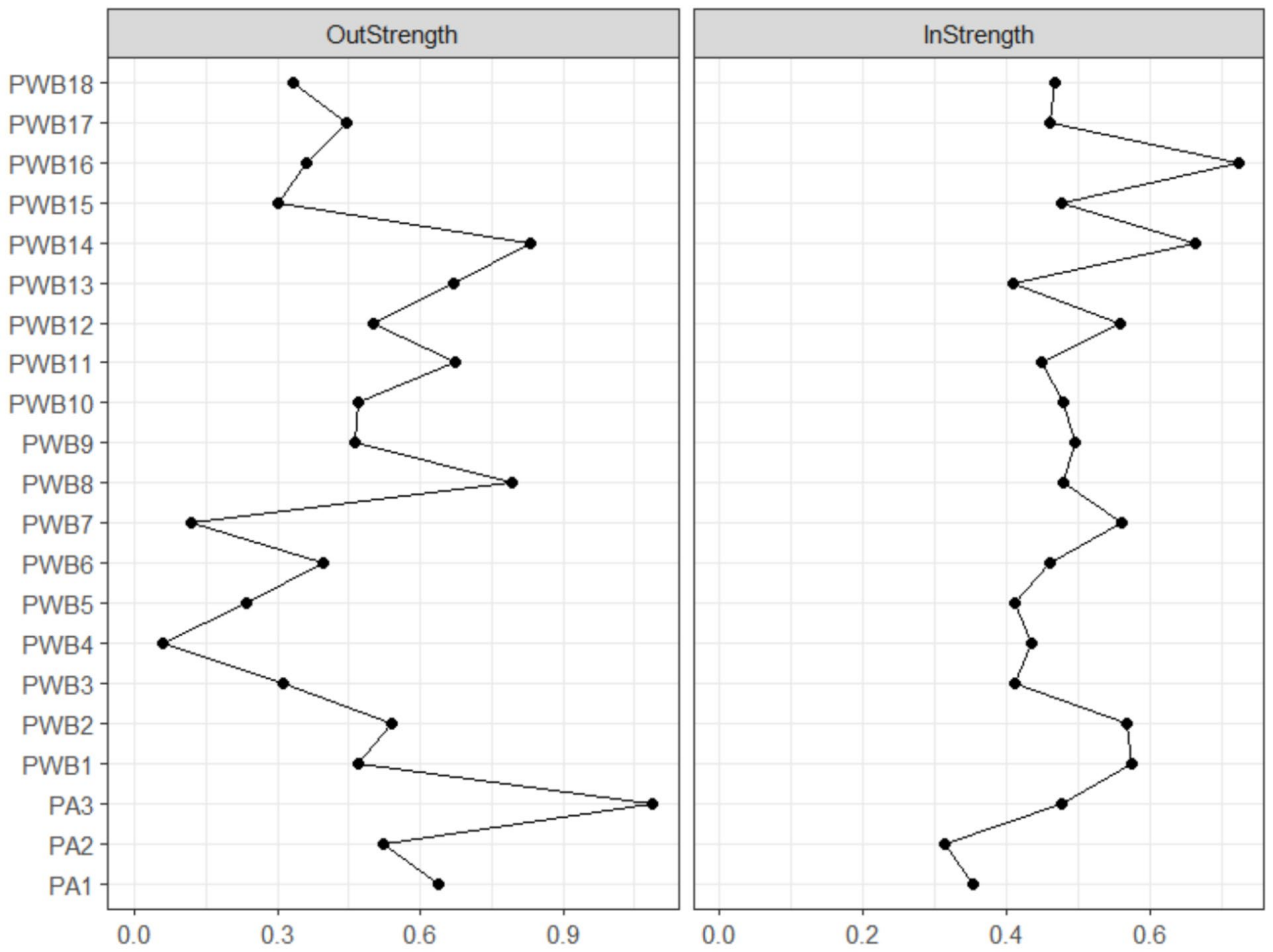
Centrality indicators of the cross-lagged panel network of physical activity and psychological well-being.

The results of the centrality analysis of the cross-lagged panel network are shown in Figure 5. In terms of predictive influence, the nodes with the highest outward expected influence (OEI) were “exercise frequency” (PA3, OEI = 1.088) and “meaning in life” (PWB14, OEI = 0.830), indicating that these two indicators have a broad predictive effect on other variables within the system. Specifically, PA3, as the core behavioral indicator of physical activity, demonstrates a significant driving potential for the psychological well-being system. Meanwhile, PWB14 reflects the central role of meaning-seeking in promoting the positive development of the system. In terms of receiving influence, “self-affirmation” (PWB16, IEI = 0.723) and “meaning in life” (PWB14, IEI = 0.662) exhibited the highest inward expected influence (IEI), suggesting that they are more likely to be influenced by other variables and are important targets of influence within the network. Notably, PWB14 ranked highly on both OEI and IEI, indicating that it holds both a driving and sensitive role, and it may play a key bridging role in the dynamic interaction between physical activity and psychological well-being. The centrality indicators for all nodes can be found in Supplementary Table S8.

The stability test of the cross-lagged panel network showed that the correlation stability coefficient (CS coefficient) for the network edge weights was 0.283, which meets the acceptable standard (CS > 0.25), confirming that the network structure estimates have basic reliability. The detailed Bootstrap confidence intervals are provided in the supplementary materials.

## 4 Discussion

### 4.1 Dynamic characteristics of the cross-sectional network structure

The age distribution of participants in this study indicated that more than 50% of the participants were between the ages of 60 and 70 years. This age group may correspond to the typical retirement age in China, where the legal retirement age is 60 for men and 55 for women. At the T1 time point, the strongest association within the psychological well-being dimensions was observed between “personal growth” (PWB7) and “openness to new experiences” (PWB8), which aligns closely with psychological well-being theory. This suggests that, in old age, continuous learning and cognitive flexibility remain core elements of psychological flourishing (48). In the cross-domain connections, the weak link between “exercise duration per session” (PA2) and “social support availability” (PWB12) may be limited in strength but hints that physical activity could provide emotional support resources for older adults individuals through social interaction mechanisms (49). This aligns with the social support theory which posits that participation in activities can promote social connections (50). By T2, the core association in the network shifted to the high covariance between “autonomous value” (PWB2) and “self-assurance” (PWB3), indicating that, over time, the unity and stability of an individual’s internal value system became the center of the psychological well-being system (51). This result supports the core position of autonomy as a fundamental psychological need in self-determination theory (18), and also reflects the increasing importance that older adults individuals in China place on internal value recognition as they adapt to aging (52). At the same time, a preliminary connection emerged in the cross-domain associations between “exercise intensity” (PA1) and “sense of personal growth” (PWB9), suggesting that the intensity of physical activity may begin to be perceived by individuals as a positive experience that promotes self-development (53). This also indicates that high-intensity exercise may have potential value in fostering psychological growth (54).

Of particular note is the significant shift in the cross-domain associations from T1 to T2: from the initial weak connection between physical activity and social support, to a substantial association between exercise intensity and a sense of personal growth. This evolution reveals a potential stage-specific characteristic in the internal mechanisms by which physical activity influences psychological well-being—during the initial phase, it primarily works through enhancing social support networks, and as participation deepens, it increasingly promotes psychological well-being by stimulating personal growth experiences (55). This finding has important implications for practical interventions: different strategies should be adopted at various stages in promoting the physical and mental health of older adults individuals. In the early stages, the focus should be on establishing social support through group activities, while in the later stages, attention should shift to how personalized exercise prescriptions can stimulate individual growth experiences, thereby achieving more precise and effective health promotion (56, 57).

### 4.2 Evolution of centrality indicators in the cross-sectional network

The centrality analysis of the cross-sectional network revealed the temporal evolution of key driving factors in the physical and mental system of older adults individuals. At the T1 time point, “life satisfaction” (PWB18) exhibited the highest centrality, which aligns with the stage characteristics of psychological adaptation in old age (58). According to psychosocial development theory, the primary developmental task in old age is achieving self-integration, and life satisfaction is a core indicator of an individual’s accomplishment of this task (59). Early in the aging process, individuals are more likely to establish the meaning of life by reviewing and evaluating their past life experiences, making life satisfaction a key node influencing the entire psychological well-being system (60).

By the T2 time point, the most influential node in the network shifted to “growth mindset” (PWB7), reflecting a shift in the focus of psychological adaptation in older adults individuals (61). As individuals gradually complete the integration of the past, their attention may shift from “retrospective evaluation” to “prospective development.” According to the multidimensional model of psychological well-being, personal growth is a fundamental element throughout life, and maintaining a growth mindset in old age is particularly important (62). It helps older adults individuals maintain a positive mindset and adaptability when facing physiological decline and changes in social roles (63). This shift also supports the theory of successful aging in the SOC (Selection, Optimization, and Compensation) model, which suggests that older adults individuals adjust the allocation of psychological resources, shifting the focus from simple satisfaction to a more vibrant sense of growth (64).

The temporal changes in centrality indicators reveal the dynamic characteristics of the psychological adaptation process in older adults individuals: from the initial phase of integration and acceptance to the later phase of development and growth (65). This finding has important implications for practical interventions, suggesting that when designing health promotion programs for the older adults, differentiated strategies should be adopted based on the psychological needs at different stages. In the early stages of the program, the focus should be on helping older adults individuals establish a positive life review and acceptance (66). In subsequent stages, the emphasis should shift to cultivating a mindset of continuous learning and growth, thereby achieving more precise and effective psychological enhancement (67).

### 4.3 Temporal relationships in the cross-lagged panel network

The cross-lagged panel network analysis revealed the complex temporal interaction mechanisms between physical activity and psychological well-being. First, within the physical activity system, the predictive effect of “exercise duration” (PA2) on “exercise frequency” (PA3) was the most significant, which aligns with the theory of behavioral consistency (68). Empirical research shows that once older adults individuals invest longer durations in a single exercise session, they are more likely to develop regular exercise habits (69). People tend to maintain established behavior patterns to reduce decision-making costs. In the context of community activities in China, longer exercise durations often signify deeper social interactions and skill mastery, which together help sustain exercise habits (70). Second, within the psychological well-being system, the predictive path from “self-acceptance” (PWB17) to “self-affirmation” (PWB16) profoundly reflects the internal mechanisms between dimensions in the psychological well-being model (71). Empirical studies show that older adults individuals’ acceptance of their limitations is fundamental to building a stable sense of self-worth (72). Especially in the context of traditional Chinese culture, which emphasizes the concept of “knowing one’s destiny” (Zhi Tian Ming), older adults individuals’ full acceptance of themselves is more likely to translate into positive self-evaluation (73). Third, the cross-domain prediction from “exercise frequency” (PA3) to “autonomous values” (PWB2) empirically confirms the prospective association of physical activity on psychological development. This finding is consistent with the core proposition of self-determination theory, which suggests that the satisfaction of basic psychological needs has domain-general characteristics (18). Observations in the field show that older adults individuals who frequently participate in group activities such as Tai Chi or square dancing not only improve their physical fitness through skill mastery and rule adherence, but also enhance their ability to make independent decisions and value judgments (74, 75). Finally, the significant autoregressive effect of “exercise duration” (PA2). Research indicates that exercise duration, as a specific and measurable behavior indicator, is more likely to form stable behavior patterns compared to other physical activity dimensions (76). This characteristic is particularly evident in the context of Chinese health culture, which emphasizes “persistence” in maintaining a consistent and sustained approach to health practices (77).

These findings collectively outline a virtuous cycle for promoting the physical and mental health of older adults individuals: by establishing stable exercise patterns, psychological basic needs are met, which in turn promotes the overall development of psychological well-being. This mechanism provides an important theoretical foundation and practical guidance for designing phased, multi-level health intervention programs for the older adults.

### 4.4 Dynamic significance of cross-lagged network centrality

The cross-lagged network centrality analysis further revealed the dynamic roles and functional positioning of various elements within the physical activity and psychological well-being systems. The results showed that “exercise frequency” (PA3) exhibited the strongest outward predictive influence (OEI = 1.088), which dynamically supports the core concept of habit formation theory (78). Empirical research indicates that regular participation in physical activity is not only an indicator of healthy behavior but also a key driving force that initiates the positive cycle of mind and body (79). In the life context of older adults individuals in China, fixed weekly group activities such as square dancing and Tai Chi help establish a regular life rhythm and social participation pattern, providing stable external input to the entire psychological well-being system (80).

A particularly important aspect to explore is the special position of “meaning in life” (PWB14) within the system, as it holds both a high outward predictive influence (OEI = 0.830) and a significant inward influence (IEI = 0.662). This dual characteristic aligns with the core concept of Frankl’s meaning-centered therapy theory, which emphasizes that the search for meaning is both a goal of psychological development and a process that promotes growth (81). In the Chinese cultural context, older adults individuals often seek meaning in life through various avenues, such as intergenerational transmission and community participation, with regular physical activity providing a platform and environment for realizing these meanings (82). The bridging role of PWB14 suggests that it may be an important hub connecting physical activity and the dimensions of psychological well-being—both transforming the benefits of physical activity into deeper psychological experiences and further promoting the depth and continuity of exercise participation through an enhanced sense of meaning (83). On the other hand, “self-affirmation” (PWB16), as the most inwardly influenced node (IEI = 0.723), reflects the acceptance characteristics of older adults individuals in the mind–body interaction process. In the identity transition that Chinese older adults individuals experience after retirement, the establishment of self-affirmation often depends on continuous external recognition and achievement experiences, and physical activity provides a platform for such validation (84).

The distribution characteristics of these centrality indicators provide important insights for designing targeted health promotion programs for the older adults: enhancing meaning in life should be prioritized as the core intervention target. By creating physical activity programs that are rich in meaning, it is possible to simultaneously activate older adults individuals’ behavioral engagement and psychological development, thereby establishing a virtuous cycle where physical activity and psychological well-being mutually promote each other (85).

### 4.5 Hybrid human–AI society

Finally, when considering the future social context of aging, it is important to recognize that older adults are increasingly likely to live in a hybrid society in which human relationships are intertwined with interactions with AI agents, including systems based on large language models. Recent studies indicate that such agents can display culturally patterned values, interpersonal styles, and biases, and that their behavior may vary across cultural settings (86). If AI companions, digital health coaches, or service robots become integrated into older adults’ daily lives, they may alter the pathways identified in this study—for example, by shaping access to physical activity opportunities, providing new forms of social and emotional support, or reinforcing particular value orientations related to autonomy and meaning in life (87). Future longitudinal research should therefore examine how AI-mediated interactions interface with the network of physical activity and psychological well-being, and whether differences in the design and cultural alignment of AI systems strengthen, weaken, or qualitatively change the dynamic associations observed here for Chinese older adults.

## 5 Conclusion

This study, through cross-lagged panel network analysis, systematically revealed the dynamic interaction mechanisms between physical activity and psychological well-being in older adults Chinese individuals. The research found that physical activity not only has temporal dependencies among its internal components but also promotes psychological development by enhancing core psychological well-being dimensions, such as autonomous values. Additionally, improvements in psychological well-being can, in turn, support the continuity of physical activity participation. Both cross-sectional and cross-lagged network analyses indicate that the mind–body system in the older adults evolves from social support to personal growth, with meaning in life playing a key bridging role in connecting behavioral engagement and psychological development. These findings offer new perspectives for understanding the intrinsic mechanisms of promoting physical and mental health in older adults individuals. They suggest that future interventions should focus on creating physical activity programs rich in meaning, adopting differentiated strategies at different stages, in order to more effectively achieve the synergistic enhancement of both physical activity and psychological well-being in the older adults.

## 6 Contributions

### 6.1 Theoretical significance

This study, by introducing the cross-lagged panel network analysis method, overcomes the limitations of traditional variable-centered paradigms and, for the first time, reveals the multidimensional and bidirectional interaction paths between physical activity and psychological well-being in older adults individuals within a dynamic system. From a theoretical perspective, this research not only validates the core role of basic psychological needs in the mind–body connection, as proposed in self-determination theory (18), but also expands the explanatory scope of the successful aging model in understanding the behavioral-psychological interaction mechanism (88). The study highlights the bridging role of meaning in life within the system, providing a new theoretical perspective for understanding the psychological adaptation process of older adults Chinese individuals from social connection to value realization. Furthermore, it provides empirical evidence for the development of psychological health frameworks for the older adults in cross-cultural contexts.

### 6.2 Practical significance

On a practical level, this study provides scientific evidence for designing targeted, phased health promotion programs for the older adults. The results suggest that in the early stages, social support should be strengthened through group physical activities, while later stages should focus on stimulating personal growth experiences (89). This finding aids communities and service organizations in developing more targeted mind–body integration intervention strategies. Moreover, with exercise frequency and “meaning in life identified as key driving nodes, the study emphasizes the importance of incorporating meaning-rich physical activity programs in public health projects, effectively enhancing participation continuity and psychological benefits. These outcomes have significant practical implications for promoting healthy aging, moving from concept to implementation, and optimizing the older adults health service system.

## 7 Research limitations and future directions

This study still has several limitations. First, although a longitudinal design was used, the data only includes two time points, which limits the ability to capture more complex nonlinear dynamic processes. Second, the sample was drawn from specific community and nursing home settings, so the findings may not be generalizable to older adults populations in rural areas or those from different cultural backgrounds. The sample may also be biased toward healthier, more socially engaged older adults, as these individuals are more likely to participate in community-based activities and institutions such as nursing homes or senior universities. This bias may limit the generalizability of the findings to older adults individuals who are less socially engaged or who face greater health challenges, such as those in rural areas or those with significant health limitations. Therefore, caution should be exercised when applying the results to the wider older adults population, particularly those in rural areas or with poorer health. Additionally, the study relied on self-reported questionnaires, including the Physical Activity Rating Scale, which is based on three items. While the reported Cronbach’s alpha for this scale was good, we acknowledge that the self-reported nature of the scale is prone to recall bias and social desirability bias. The granularity of the data is also limited compared to more objective measures, such as accelerometer-based assessments, which would provide more precise and reliable measurements of physical activity. Future research could benefit from incorporating objective activity monitoring and multidimensional assessment indicators to improve the precision of the findings. Lastly, the study did not collect data on the employment status of participants, which could potentially influence the study’s findings. Employment status may have an impact on both physical activity levels and psychological well-being, and future studies should consider collecting this information for a more comprehensive understanding of the factors at play. Finally, while the cross-lagged network analysis can reveal predictive relationships, statistical prediction does not equate to causal inference, and there is room for improvement in model stability. Moreover, the present design focuses on variables at the psychological and behavioral levels and does not incorporate biological markers, fine-grained features of the social and natural environment, or macro-level cultural indicators, which constrains our ability to characterize how processes across different levels jointly shape the development of physical activity and psychological well-being in later life.

Future research could further expand in the following areas: increasing the number of tracking time points and shortening the intervals between them, using intensive longitudinal data to capture dynamic details; expanding the sample to include more representative groups, such as rural populations and ethnic minorities, for cross-cultural comparisons; integrating physiological indicators, social networks, and other multi-level data to construct a more comprehensive explanatory framework; combining intervention experiments and case tracking to verify the causal effects and clinical applicability of key pathways in the network, thereby advancing the systematic development of older adults health promotion theories and practices. In addition, recent work on cross-scale, interdisciplinary science suggests that understanding psychological well-being requires connecting processes from micro-level biological mechanisms to meso-level social interactions and macro-level cultural and environmental dynamics (90). Building on this perspective, future studies could embed cross-lagged panel network models within multi-level data structures that link individual trajectories of physical activity and psychological well-being to information on genetic or physiological markers, neighborhood walkability and access to green space, and cultural norms surrounding aging and physical activity. Such cross-scale, interdisciplinary designs would make it possible to test whether the micro-level patterns identified in the present study are amplified, constrained, or transformed by broader social, cultural, and natural environments, thereby moving toward a more integrated understanding of psychological well-being in aging societies.

## Data Availability

The original contributions presented in the study are included in the article/supplementary material, further inquiries can be directed to the corresponding authors.
