# Supplementary material for: The dynamic relationship between physical activity and psychological well-being in Chinese older adults: a longitudinal cross-lagged panel network analysis
Source: Front Public Health. 2026 Jan 6;13:1736019. doi: 10.3389/fpubh.2025.1736019 (PMC12815800; doi:10.3389/fpubh.2025.1736019)
Supplement: Supplementary file 1 [file Table_1.DOCX]

Supplementary Material

# Physical Activity Rating Scale Response Anchors

The Physical Activity Rating Scale used in this study measures physical activity across three dimensions: exercise intensity, exercise frequency, and exercise duration. The scale uses a five-point Likert scale for each dimension, with the following response anchors:

Intensity:

1 = Very low intensity (e.g., very light walking or sedentary behavior)

2 = Low intensity (e.g., light walking or minimal physical exertion)

3 = Moderate intensity (e.g., brisk walking, moderate exercise)

4 = High intensity (e.g., fast walking, vigorous exercise)

5 = Very high intensity (e.g., intense physical exertion, running, or competitive sports)

Duration:

1 = Less than 10 minutes

2 = 10-20 minutes

3 = 21-30 minutes

4 = 31-45 minutes

5 = More than 45 minutes

Frequency:

1 = Never (0 times per week)

2 = Rarely (1-2 times per week)

3 = Occasionally (3-4 times per week)

4 = Frequently (5-6 times per week)

5 = Daily (7 or more times per week)

# Supplementary Figures and Tables

## Supplementary Tables

**Table S-1** Confirmatory Factor Analysis Fit Indices for the Psychological Well-Being Scale (T1 and T2)

|  | ^2^/df | RMSEA | SRMR | CFI | TLI |
| --- | --- | --- | --- | --- | --- |
| T1 | 1.140 | 0.012 | 0.012 | 0.999 | 0.999 |
| T2 | 1.481 | 0.022 | 0.014 | 0.994 | 0.993 |

**Table S-2** Cronbach's α Coefficients for Each Dimension of the Psychological Well-Being Scale (T1 and T2)

| Dimension | T1 Cronbach's α | T2 Cronbach's α |
| --- | --- | --- |
| Autonomy | 0.931 | 0.872 |
| Environmental Mastery | 0.922 | 0.866 |
| Personal Growth | 0.934 | 0.868 |
| Positive Relationships with Others | 0.918 | 0.851 |
| Life Purpose | 0.926 | 0.877 |
| Self-Acceptance | 0.928 | 0.872 |

**Table S-3** T1 Cross-Sectional Network Edge Weights (Top 30)

| Rank | Node |  | Node | Correlation Coefficient |
| --- | --- | --- | --- | --- |
| 1 | T1_PWB7 | - | T1_PWB8 | 0.488 |
| 2 | T1_PWB13 | - | T1_PWB14 | 0.458 |
| 3 | T1_PWB10 | - | T1_PWB12 | 0.449 |
| 4 | T1_PWB4 | - | T1_PWB6 | 0.445 |
| 5 | T1_PWB2 | - | T1_PWB3 | 0.445 |
| 6 | T1_PWB10 | - | T1_PWB11 | 0.437 |
| 7 | T1_PWB1 | - | T1_PWB2 | 0.437 |
| 8 | T1_PWB8 | - | T1_PWB9 | 0.419 |
| 9 | T1_PWB13 | - | T1_PWB15 | 0.407 |
| 10 | T1_PWB4 | - | T1_PWB5 | 0.407 |
| 11 | T1_PWB5 | - | T1_PWB6 | 0.403 |
| 12 | T1_PWB14 | - | T1_PWB15 | 0.401 |
| 13 | T1_PA2 | - | T1_PA3 | 0.393 |
| 14 | T1_PWB1 | - | T1_PWB3 | 0.389 |
| 15 | T1_PWB7 | - | T1_PWB9 | 0.381 |
| 16 | T1_PWB16 | - | T1_PWB17 | 0.359 |
| 17 | T1_PA1 | - | T1_PA2 | 0.354 |
| 18 | T1_PWB11 | - | T1_PWB12 | 0.341 |
| 19 | T1_PA1 | - | T1_PA3 | 0.339 |
| 20 | T1_PWB11 | - | T1_PWB17 | 0.052 |
| 21 | T1_PWB5 | - | T1_PWB15 | 0.050 |
| 22 | T1_PA2 | - | T1_PWB12 | 0.048 |
| 23 | T1_PA3 | - | T1_PWB1 | 0.036 |
| 24 | T1_PWB10 | - | T1_PWB16 | 0.034 |
| 25 | T1_PA3 | - | T1_PWB17 | 0.033 |
| 26 | T1_PA1 | - | T1_PWB9 | 0.033 |
| 27 | T1_PA1 | - | T1_PWB16 | 0.033 |
| 28 | T1_PA2 | - | T1_PWB6 | 0.032 |
| 29 | T1_PWB2 | - | T1_PWB9 | 0.032 |

**Table S-4** T2 Cross-Sectional Network Edge Weights (Top 30)

| Rank | Node |  | Node | Correlation Coefficient |
| --- | --- | --- | --- | --- |
| 1 | T2_PWB2 | - | T2_PWB3 | 0.393 |
| 2 | T2_PWB7 | - | T2_PWB9 | 0.388 |
| 3 | T2_PA1 | - | T2_PA3 | 0.384 |
| 4 | T2_PWB7 | - | T2_PWB8 | 0.375 |
| 5 | T2_PWB4 | - | T2_PWB6 | 0.370 |
| 6 | T2_PWB13 | - | T2_PWB14 | 0.367 |
| 7 | T2_PWB10 | - | T2_PWB11 | 0.366 |
| 8 | T2_PWB14 | - | T2_PWB15 | 0.359 |
| 9 | T2_PWB4 | - | T2_PWB5 | 0.357 |
| 10 | T2_PA2 | - | T2_PA3 | 0.353 |
| 11 | T2_PWB13 | - | T2_PWB15 | 0.350 |
| 12 | T2_PWB10 | - | T2_PWB12 | 0.345 |
| 13 | T2_PWB1 | - | T2_PWB2 | 0.335 |
| 14 | T2_PWB5 | - | T2_PWB6 | 0.327 |
| 15 | T2_PWB1 | - | T2_PWB3 | 0.324 |
| 16 | T2_PWB16 | - | T2_PWB17 | 0.316 |
| 17 | T2_PA1 | - | T2_PA2 | 0.299 |
| 18 | T2_PWB8 | - | T2_PWB9 | 0.288 |
| 19 | T2_PWB11 | - | T2_PWB12 | 0.262 |
| 20 | T2_PWB1 | - | T2_PWB11 | 0.085 |
| 21 | T2_PWB7 | - | T2_PWB13 | 0.080 |
| 22 | T2_PWB2 | - | T2_PWB12 | 0.070 |
| 23 | T2_PA1 | - | T2_PWB9 | 0.061 |
| 24 | T2_PWB2 | - | T2_PWB6 | 0.059 |
| 25 | T2_PWB7 | - | T2_PWB12 | 0.058 |
| 26 | T2_PWB11 | - | T2_PWB17 | 0.057 |
| 27 | T2_PWB2 | - | T2_PWB8 | 0.051 |
| 28 | T2_PWB11 | - | T2_PWB16 | 0.049 |
| 29 | T2_PWB1 | - | T2_PWB5 | 0.047 |
| 30 | T2_PWB6 | - | T2_PWB8 | 0.046 |

**Table S-5** T1 Cross-Sectional Network Centrality Indicators (Expected Influence)

| Rank | Node | Expected Influence |
| --- | --- | --- |
| 1 | PA1 | -2.596 |
| 2 | PA2 | -0.023 |
| 3 | PA3 | -1.466 |
| 4 | PWB1 | 0.737 |
| 5 | PWB2 | 0.585 |
| 6 | PWB3 | -0.723 |
| 7 | PWB4 | 0.053 |
| 8 | PWB5 | 0.258 |
| 9 | PWB6 | 0.240 |
| 10 | PWB7 | 0.287 |
| 11 | PWB8 | 1.219 |
| 12 | PWB9 | 0.136 |
| 13 | PWB10 | 0.740 |
| 14 | PWB11 | -0.723 |
| 15 | PWB12 | 0.059 |
| 16 | PWB13 | 0.024 |
| 17 | PWB14 | 1.262 |
| 18 | PWB15 | -0.871 |
| 19 | PWB16 | -1.208 |
| 20 | PWB17 | 0.269 |
| 21 | PWB18 | 1.740 |

**Table S-6** T2 Cross-Sectional Network Centrality Indicators (Expected Influence)

| Rank | Node | Expected Influence |
| --- | --- | --- |
| 1 | PA1 | -1.157 |
| 2 | PA2 | -3.318 |
| 3 | PA3 | 0.429 |
| 4 | PWB1 | -0.455 |
| 5 | PWB2 | 1.398 |
| 6 | PWB3 | 0.311 |
| 7 | PWB4 | -0.204 |
| 8 | PWB5 | 0.865 |
| 9 | PWB6 | -0.174 |
| 10 | PWB7 | 1.571 |
| 11 | PWB8 | -0.910 |
| 12 | PWB9 | -0.219 |
| 13 | PWB10 | 0.073 |
| 14 | PWB11 | 0.158 |
| 15 | PWB12 | -0.093 |
| 16 | PWB13 | 0.377 |
| 17 | PWB14 | 0.379 |
| 18 | PWB15 | 0.186 |
| 19 | PWB16 | -0.295 |
| 20 | PWB17 | 0.641 |
| 21 | PWB18 | 0.438 |

**Table S-7** Cross-Lagged Network Analysis Regression Coefficients (Top 30)

| Rank | Node | → | Node | Regression Type | Regression Coefficient |
| --- | --- | --- | --- | --- | --- |
| 1 | PA2 | → | PA3 | Cross-Lagged | 0.187 |
| 2 | PWB17 | → | PWB16 | Cross-Lagged | 0.184 |
| 3 | PA3 | → | PWB2 | Cross-Lagged | 0.166 |
| 4 | PA2 | → | PA2 | Autoregressive | 0.152 |
| 5 | PA1 | → | PA | Autoregressive | 0.151 |
| 6 | PWB11 | → | PWB14 | Cross-Lagged | 0.132 |
| 7 | PA3 | → | PWB1 | Cross-Lagged | 0.129 |
| 8 | PWB14 | → | PWB14 | Autoregressive | 0.129 |
| 9 | PWB8 | → | PWB2 | Cross-Lagged | 0.123 |
| 10 | PWB14 | → | PWB8 | Cross-Lagged | 0.121 |
| 11 | PA3 | → | PWB9 | Cross-Lagged | 0.110 |
| 12 | PWB1 | → | PWB5 | Cross-Lagged | 0.110 |
| 13 | PWB11 | → | PWB13 | Cross-Lagged | 0.109 |
| 14 | PWB15 | → | PWB9 | Cross-Lagged | 0.104 |
| 15 | PA3 | → | PWB3 | Cross-Lagged | 0.101 |
| 16 | PWB18 | → | PWB16 | Cross-Lagged | -0.098 |
| 17 | PWB | → | PWB16 | Cross-Lagged | 0.096 |
| 18 | PWB14 | → | PWB7 | Cross-Lagged | 0.095 |
| 19 | PWB10 | → | PWB8 | Cross-Lagged | 0.094 |
| 20 | PWB13 | → | PWB12 | Cross-Lagged | 0.094 |
| 21 | PWB8 | → | PWB3 | Cross-Lagged | 0.094 |
| 22 | PWB16 | → | PWB10 | Cross-Lagged | 0.092 |
| 23 | PA3 | → | PA2 | Cross-Lagged | 0.092 |
| 24 | PWB9 | → | PWB17 | Cross-Lagged | 0.087 |
| 25 | PWB15 | → | PWB14 | Cross-Lagged | -0.086 |
| 26 | PWB6 | → | PWB4 | Cross-Lagged | 0.084 |
| 27 | PA3 | → | PA3 | Autoregressive | 0.083 |
| 28 | PWB14 | → | PWB1 | Cross-Lagged | 0.083 |
| 29 | PWB10 | → | PWB12 | Cross-Lagged | 0.082 |
| 30 | PA3 | → | PWB10 | Cross-Lagged | 0.080 |

**Table S-8** Cross-Lagged Network Directed Bridge Centrality Indicators (InStrength and OutStrength)

| node | InStrength | node | OutStrength |
| --- | --- | --- | --- |
| PA1 | 0.354 | PA1 | 0.640 |
| PA2 | 0.313 | PA2 | 0.522 |
| PA3 | 0.477 | PA3 | 1.088 |
| PWB1 | 0.574 | PWB1 | 0.469 |
| PWB2 | 0.567 | PWB2 | 0.541 |
| PWB3 | 0.412 | PWB3 | 0.314 |
| PWB4 | 0.434 | PWB4 | 0.060 |
| PWB5 | 0.411 | PWB5 | 0.235 |
| PWB6 | 0.461 | PWB6 | 0.397 |
| PWB7 | 0.561 | PWB7 | 0.120 |
| PWB8 | 0.478 | PWB8 | 0.792 |
| PWB9 | 0.496 | PWB9 | 0.462 |
| PWB10 | 0.479 | PWB10 | 0.469 |
| PWB11 | 0.448 | PWB11 | 0.673 |
| PWB12 | 0.558 | PWB12 | 0.502 |
| PWB13 | 0.410 | PWB13 | 0.668 |
| PWB14 | 0.662 | PWB14 | 0.830 |
| PWB15 | 0.478 | PWB15 | 0.300 |
| PWB16 | 0.723 | PWB16 | 0.360 |
| PWB17 | 0.461 | PWB17 | 0.445 |
| PWB18 | 0.467 | PWB18 | 0.334 |

## Supplementary Figures


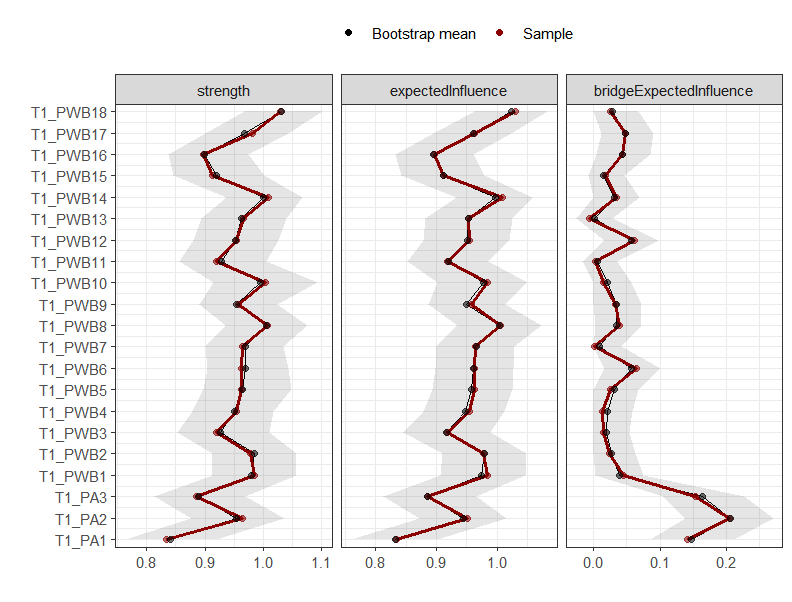


**Figure S-1** Bootstrap Confidence Intervals for Edge Weights in T1 Cross-Sectional Network

Note: This figure displays the Bootstrap confidence intervals for the edge weights in the T1 cross-sectional network. The confidence intervals help assess the stability and reliability of the network estimates.


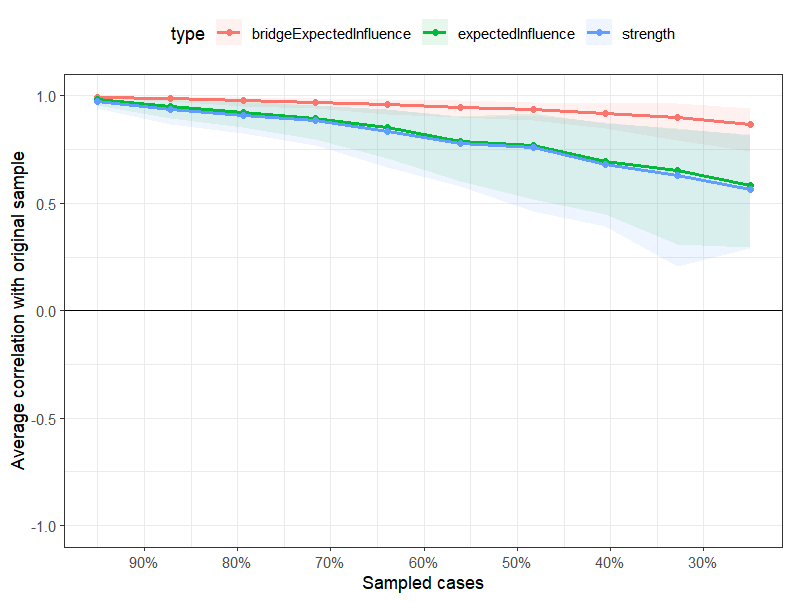


**FigurS-2** Bootstrap Results for Centrality Indicators in T1 Cross-Sectional Networke

Note: This figure illustrates the Bootstrap results for the centrality indicators in the T1 cross-sectional network. The results reflect the stability and reliability of the centrality measures, including expected influence and other relevant metrics.


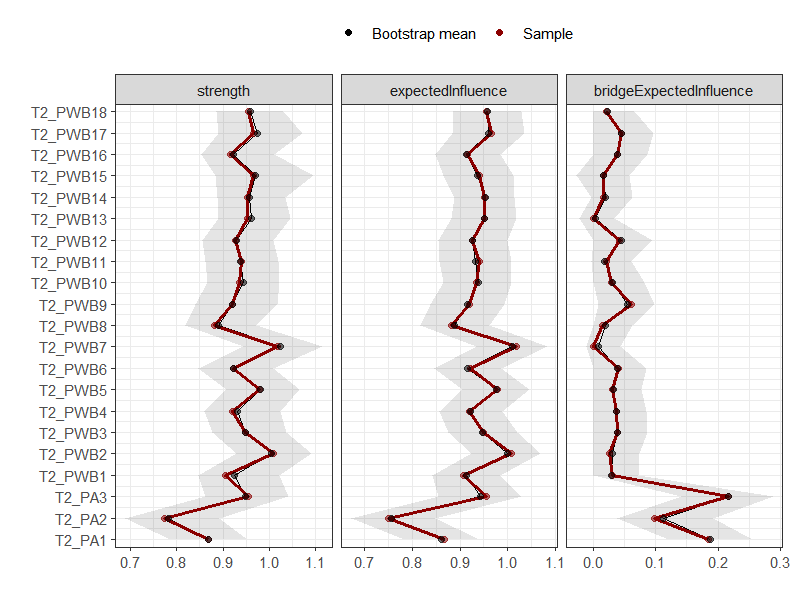


**Figure S-3** Bootstrap Confidence Intervals for Edge Weights in T2 Cross-Sectional Network

Note: This figure displays the Bootstrap confidence intervals for the edge weights in the T2 cross-sectional network. These intervals are used to assess the stability and accuracy of the edge weight estimates in the network.


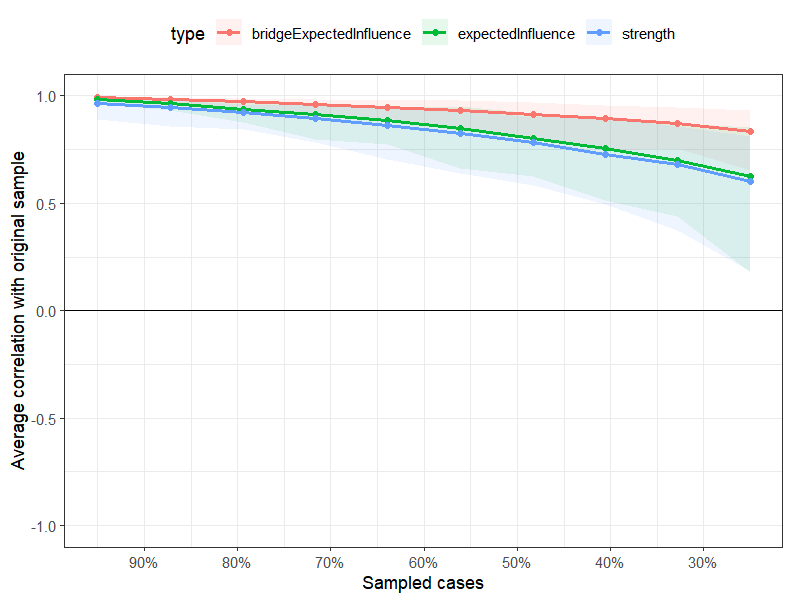


**Figure S-4** Bootstrap Results for Centrality Indicators in T2 Cross-Sectional Network

Note: This figure presents the Bootstrap results for the centrality indicators in the T2 cross-sectional network. These results show the stability and reliability of the centrality measures, including expected influence and other relevant metrics.


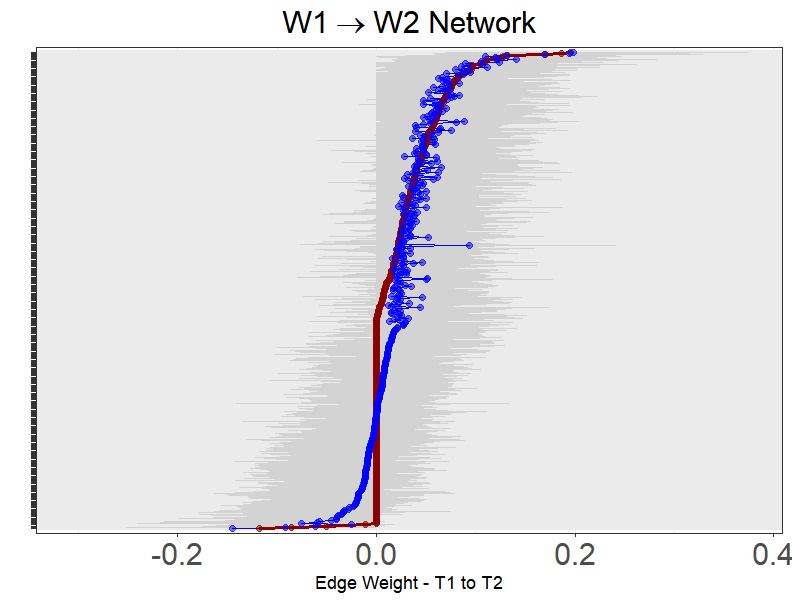


**Figure S-5** Bootstrap Confidence Intervals for Edge Weights in the Cross-Lagged Panel Network

Note: This figure displays the Bootstrap confidence intervals for the edge weights in the cross-lagged panel network. The confidence intervals help assess the stability and reliability of the edge weight estimates in the network.
